# Comparative Outcomes of a Pediatric Walking Rehabilitation Robot Combined With Repetitive Transcranial Magnetic Stimulation in Children With Spastic Cerebral Palsy: Protocol for a Prospective Observational Study

**DOI:** 10.2196/93798

**Published:** 2026-06-29

**Authors:** Weiyi Huang, Cuihua Shan, Xiaoyu Shen, Jianguo Zhong

**Affiliations:** 1 School of Laboratory Medicine Chengdu Medical College Chengdu, Sichuan China; 2 Rehabilitation Department China National Nuclear Corporation 416 Hospital The 2nd Affiliated Hospital of Chengdu Medical College Chengdu, Sichuan China

**Keywords:** cerebral palsy, CP, rehabilitation robot, repetitive transcranial magnetic stimulation, rTMS, rehabilitation medicine

## Abstract

**Background:**

Cerebral palsy (CP) is a prevalent neurodevelopmental disorder in children, often leading to long-term motor impairments. Rehabilitation robotics has emerged as a promising approach in pediatric neurorehabilitation, offering precise and repetitive motor training. Repetitive transcranial magnetic stimulation (rTMS), a noninvasive neuromodulation technique, has shown potential in modulating cortical excitability and improving motor function in children with CP.

**Objective:**

This study aims to evaluate the association between a combined intervention using a pediatric gait rehabilitation robot and rTMS in children with spastic CP.

**Methods:**

This prospective observational study will enroll 108 children with spastic CP who meet the inclusion criteria. Participants will be allocated to either an intervention group (robot-assisted gait training combined with rTMS) or a comparison group (conventional rehabilitation therapy) based on their guardians’ preference after standardized information provision. The assignment is nonrandomized and will be documented prospectively. Interventions will be delivered 5 times per week for 8 weeks. Outcomes will be assessed at baseline, week 4, and week 8 using 3D gait analysis, neuromuscular electromyography, the Gross Motor Function Measure-88, and the Modified Ashworth Scale. Comparative analyses will be conducted to evaluate the outcomes of the combined intervention relative to standard rehabilitation. Findings are expected to provide evidence supporting integrated neuromodulation and robotic therapy in pediatric CP rehabilitation.

**Results:**

This study was funded in 2024. Study recruitment began in January 2025, and as of June 2026, enrollment is ongoing; exact recruitment numbers are being verified with the research coordinator. This study has been approved by the ethics committee (YJ-2025-020-01) and supported by grants S2024094 and CDS2018Z005. Outcomes will be assessed at baseline, week 4, and week 8 via 3D gait analysis, electromyography, Gross Motor Function Measure-88, Modified Ashworth Scale. Data collection is expected to be complete in December 2027, and the data will be analyzed with SPSS. The results are expected to be published in spring 2028.

**Conclusions:**

This study evaluates the comparative outcomes associated with a pediatric gait rehabilitation robot combined with rTMS for children with spastic CP. Positive findings would suggest a preliminary association between the multimodal protocol and better motor function (including improved gait performance and reduced muscle spasticity), without implying causality. Such results would provide a new evidence-based option for pediatric spastic CP rehabilitation.

**Trial Registration:**

Chinese Clinical Trial Registry ChiCTR2500105823; https://www.chictr.org.cn/showproj.html?proj=274950

**International Registered Report Identifier (IRRID):**

DERR1-10.2196/93798

## Introduction

### Background

Cerebral palsy (CP) is one of the most common neurodevelopmental disorders in children and is characterized by permanent motor impairment resulting from nonprogressive injury to the developing brain. Epidemiological studies indicate that the prevalence of CP among children aged 1 to 6 years is approximately 2.46% in China and ranges from 0.14% to 0.32% globally [1]. Spastic CP, the predominant subtype, accounts for nearly 70% of cases [2,3]. It is characterized by hypertonia and hyperreflexia, often leading to abnormal posture and gait patterns, including limb spasticity, flexor dominance, trunk flexion, knee flexion, and scissor gait. These motor impairments frequently persist throughout childhood and may evolve with growth and development, substantially affecting functional independence and quality of life.

The pathophysiology of spastic CP is complex and multifactorial, involving cortical and subcortical injury, impaired neurodevelopment, and secondary musculoskeletal abnormalities [4]. A widely recognized mechanism is the disruption of supraspinal regulation of spinal stretch reflexes following central nervous system injury, resulting in exaggerated reflex excitability and increased muscle tone [5,6]. Current rehabilitation strategies are typically multimodal and include physical therapy, occupational therapy, pharmacological management, orthopedic surgery, and complementary approaches, such as traditional Chinese medicine. Despite these interventions, functional recovery remains challenging, particularly in children with moderate-to-severe motor impairments. Consequently, there is growing interest in innovative rehabilitation approaches capable of promoting neuroplasticity and improving motor outcomes.

Among these emerging interventions, repetitive transcranial magnetic stimulation (rTMS) has gained increasing attention as a noninvasive neuromodulation technique. By delivering pulsed magnetic stimulation to targeted cortical regions, rTMS can modulate neuronal excitability and facilitate neuroplastic reorganization [7]. Previous studies have demonstrated its therapeutic potential in various neurological and neuropsychiatric disorders, including stroke, anxiety, depression, and, increasingly, CP [8-12]. Mechanistically, rTMS is believed to regulate synaptic plasticity, restore interhemispheric balance, and enhance corticospinal tract function, particularly when applied to the primary motor cortex. Importantly, rTMS is generally well tolerated in pediatric populations and allows relatively precise cortical targeting. A recent meta-analysis of 16 randomized controlled trials reported that rTMS was associated with improvements in gross motor function (Gross Motor Function Measure [GMFM]; standardized mean difference [SMD] 0.47-0.63) and gait velocity (SMD 1.28) in children with CP [13]. Nevertheless, the optimal stimulation protocols and their integration with rehabilitation training remain insufficiently established.

Parallel to developments in neuromodulation, rehabilitation robotics has emerged as a promising technology in pediatric neurorehabilitation. Robotic systems can incorporate real-time monitoring, adaptive algorithms, and task-oriented training environments to deliver intensive, repetitive, and individualized motor rehabilitation [14,15]. Compared with conventional therapy, robot-assisted interventions offer improved movement reproducibility, precise control of training parameters, and enhanced feedback mechanisms that may facilitate motor learning. In this study, we used a pediatric gait rehabilitation robot (BFR-K-A200; Chengdu Buffalo Robotics Technology Company), which combines an exoskeleton with a wheeled walker to assist in standing, walking, and lower-limb coordination. This device provides active and passive joint assistance and may improve gait efficiency and training adaptability compared with conventional exoskeleton-based systems.

Although both robotic gait training and rTMS have demonstrated potential benefits individually, evidence regarding their combined application in pediatric spastic CP remains limited. Given their complementary therapeutic mechanisms—robot-assisted training targeting peripheral motor execution and gait relearning, and rTMS targeting central neuromodulation and cortical plasticity—their integration may produce synergistic rehabilitation effects. Therefore, this prospective observational study aims to evaluate the clinical outcomes associated with robot-assisted gait training combined with rTMS in children with spastic CP. Treatment effects will be assessed using 3D gait analysis, neuromuscular electromyography, the GMFM-88, and the Modified Ashworth Scale (MAS), with the goal of generating evidence to inform multimodal and evidence-based rehabilitation strategies for this population.

### Study Objective and Hypothesis

The primary objective of this study is to evaluate the clinical outcomes associated with a pediatric gait rehabilitation robot combined with rTMS in adolescents with spastic CP, compared with conventional rehabilitation therapy.

The primary hypothesis is that participants receiving robot-assisted gait training combined with rTMS may show greater improvements in gross motor function, gait performance, and muscle tone than those receiving conventional rehabilitation after 8 weeks of intervention. However, due to the observational design, causal inference is not possible. Specifically, changes in GMFM-88 scores from baseline to week 8 will serve as the primary end point. Secondary hypotheses are that the intervention group will exhibit superior improvements in 3D gait parameters, electromyography outcomes, and MAS scores at weeks 4 and 8.

Given the observational nature of the study, any observed between-group differences will be interpreted as associations rather than causal effects.

## Methods

### Study Design

This study is designed as a prospective observational controlled study to evaluate the clinical outcomes associated with robotic gait training combined with rTMS in adolescents with spastic CP. An observational design was selected primarily because treatment allocation is influenced by family preference and clinical decision-making in real-world rehabilitation settings. In addition, the highly visible and technology-intensive nature of robotic rehabilitation limits the feasibility of participant and therapist blinding. Nevertheless, to minimize assessment bias, outcome evaluations will be conducted by independent rehabilitation physicians who are not involved in treatment delivery and are blinded to group allocation whenever feasible.

**Figure 1:**
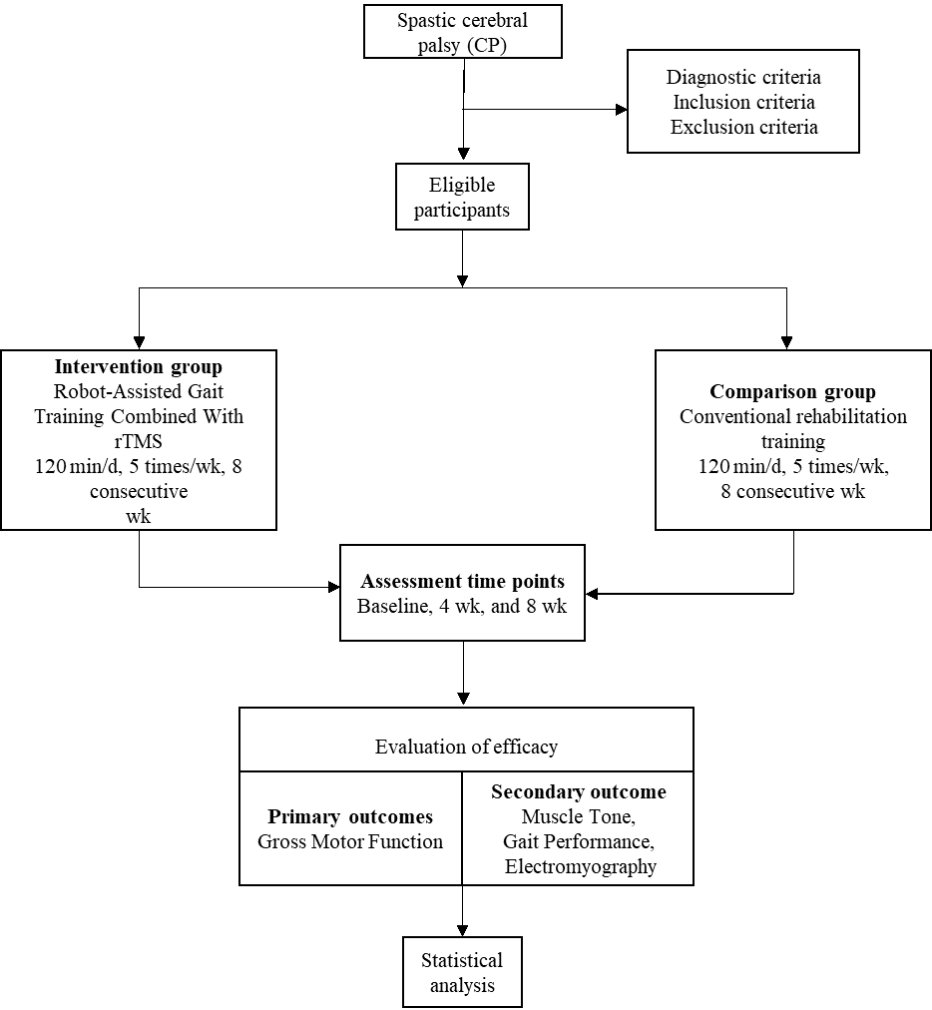
Flowchart. CP: Cerebral palsy; rTMS: repetitive transcranial magnetic stimulation.

**Figure 2:**
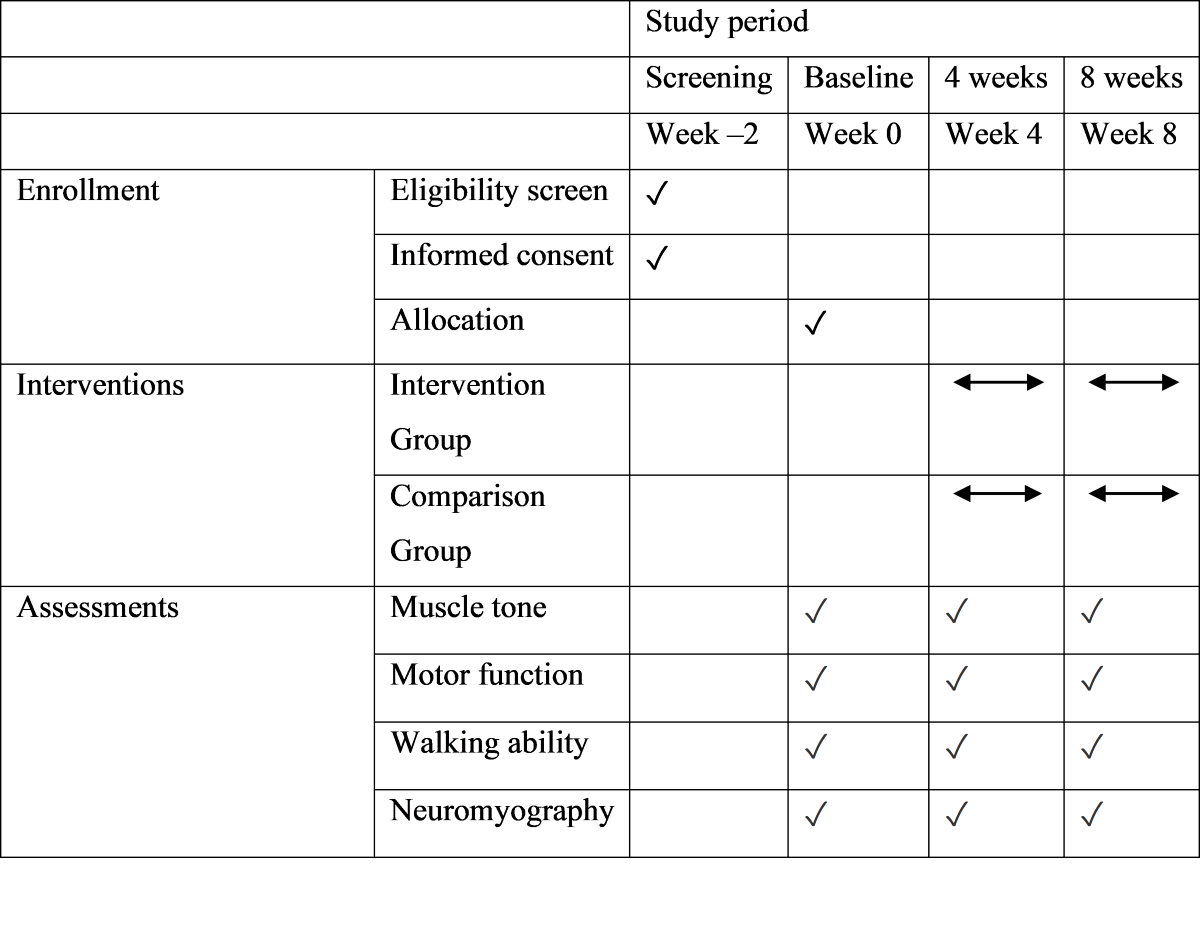
Schedule of enrollment, intervention, and assessment.

Eligible participants will be allocated to either an intervention group (robot-assisted gait training combined with rTMS) or a comparison group (conventional rehabilitation therapy) according to guardian preference after receiving standardized study information from a neutral research coordinator who is not involved in treatment administration or outcome assessment. Allocation decisions will be prospectively documented, and no randomization will be performed. Both groups will receive 5 treatment sessions per week over an 8-week intervention period. Clinical evaluations will be performed at baseline, week 4, and week 8 by trained rehabilitation physicians using standardized assessment procedures. The study flowchart is presented in Figure 1, and the schedule of enrollment, intervention, and assessment is summarized in Figure 2. This protocol was developed in accordance with the SPIRIT (Standard Protocol Items: Recommendations for Interventional Trials) guidelines and will be reported following the STROBE (Strengthening the Reporting of Observational Studies in Epidemiology) statement for observational studies [16,17]. Participant recruitment is scheduled to commence in January 2025, with data collection anticipated to be completed by December 2027.

### Participants

A total of 108 adolescents with spastic CP will be recruited from the outpatient clinic of the Department of Rehabilitation Medicine, China National Nuclear Corporation 416 Hospital. The department is a tertiary rehabilitation center providing integrated clinical, teaching, and research services with extensive experience in pediatric neurodevelopmental disorder rehabilitation.

A structured summary of all eligibility criteria is presented in Textbox 1. Inclusion criteria define the target population, while exclusion criteria identify conditions that may confound outcome interpretation or pose safety risks.

**Inclusion and exclusion criteria.**

**Inclusion criteria**

- Diagnosis of spastic cerebral palsy according to established clinical diagnostic criteria
- Aged between 13 and 18 years
- Gross Motor Function Classification System levels I-V
- Medically stable and able to participate in rehabilitation training
- Written informed consent provided by participants and/or legal guardians

**Exclusion criteria**

- Severe epilepsy or uncontrolled seizure disorder
- Severe structural brain abnormalities or progressive neurological disease
- Severe cognitive or behavioral impairment precluding cooperation
- Other serious medical conditions compromising safety or participation

### Patient and Public Involvement

Patients and the public were not involved in the design, conduct, reporting, or dissemination plans of this study because of the technical and methodological complexity of the intervention protocol.

### Criteria for Withdrawal

Participants may be withdrawn from the study under the following circumstances:

- Voluntary withdrawal of consent by participants or legal guardians
- Major protocol deviation or persistent noncompliance
- Attendance below 80% of scheduled intervention sessions
- Inability to complete key outcome assessments
- Occurrence of serious adverse events or medical complications requiring discontinuation
- Investigator judgment that continued participation may compromise participant safety or data integrity.

All withdrawals and reasons for discontinuation will be prospectively documented.

### Intervention

Participants in both groups will receive structured rehabilitation interventions over an 8-week period, consisting of 5 sessions per week.

#### Comparison Group

Participants allocated to the comparison group will receive conventional rehabilitation therapy, including resistance stretching, joint mobility training, balance training, core stability exercises, and functional electrical stimulation. Each session will last approximately 120 minutes and be delivered once daily, 5 days per week.

#### Intervention Group

Participants in the intervention group will receive robot-assisted gait training combined with rTMS.

#### Pediatric Gait Rehabilitation Robot

Robot-assisted gait training will be delivered using the pediatric gait rehabilitation robot (BFR-K-A200; Chengdu Buffalo Robotics Technology Co). This device integrates an exoskeleton with a wheeled walker and incorporates adaptive gait assistance algorithms informed by individualized movement characteristics.

Each robot-assisted training session will last 30 minutes. Participants in the intervention group will receive 2 sessions per day (one in the morning and one in the afternoon), 5 days per week, for a total of 8 weeks (ie, 80 sessions). The total daily intervention time for robot-assisted training is 60 minutes. Training intensity and gait assistance parameters will be individualized according to participant tolerance and rehabilitation needs.

#### Repetitive Transcranial Magnetic Stimulation

**Figure 3:**
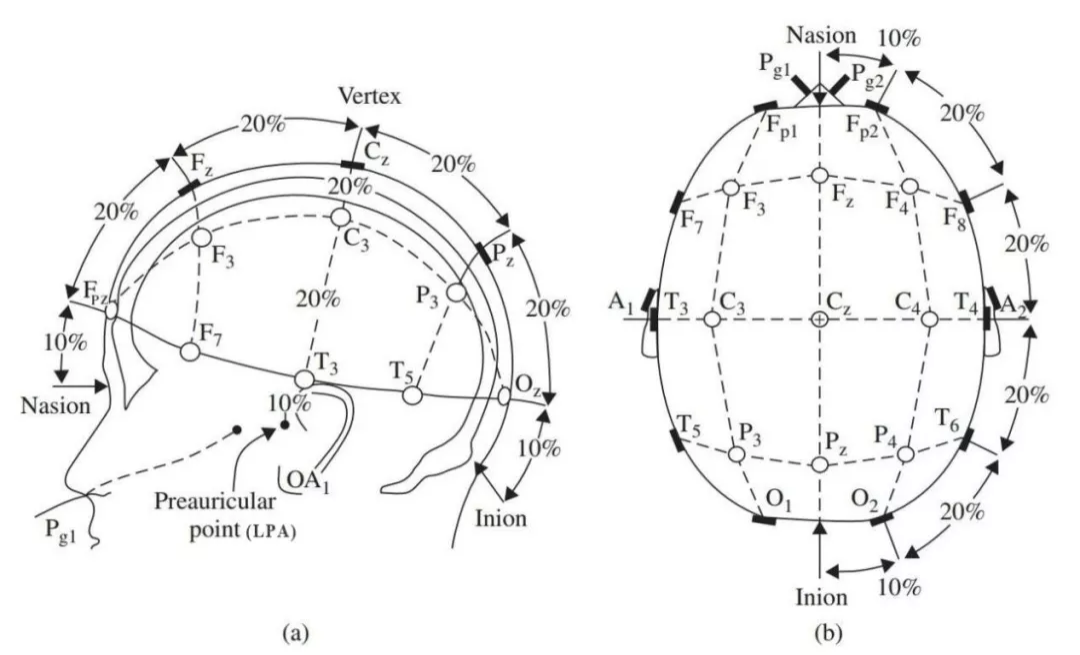
The international 10-20 electroencephalography system.

rTMS will be delivered using a figure-of-eight coil, targeting the primary motor cortex (M1) according to the international 10-20 electroencephalography positioning system (Figure 3). Before the first rTMS session, each participant’s resting motor threshold will be determined as the minimum stimulus intensity required to elicit a motor-evoked potential of ≥50 μV in the contralateral first dorsal interosseous muscle in at least 5 of 10 consecutive trials. rTMS will be delivered at 90% of resting motor threshold. Each session consists of stimulation over the affected hemisphere M1 at 5 Hz, with a total of 1200 pulses delivered in a pattern of 2 seconds of continuous stimulation followed by 8 seconds of rest. This is followed by stimulation over the unaffected hemisphere M1 at 1 Hz, with a total of 900 pulses delivered in a pattern of 1 second of continuous stimulation followed by 2 seconds of rest. Total session duration is approximately 30 minutes. rTMS will be administered twice daily, 5 days per week.

#### Concomitant Therapies

During the 8-week intervention period, participants in both groups will be asked not to receive any rehabilitation therapies outside the study protocol, as this would confound outcome interpretation. Any deviation from this requirement will be prospectively documented. Participants who receive substantial additional therapies (eg, more than 3 sessions) will be excluded from the per-protocol analysis but retained in the intention-to-treat analysis.

#### Safety Monitoring

Safety procedures will be implemented throughout all interventions, including joint screening, warm-up and cooldown exercises, equipment inspection, and continuous monitoring for adverse reactions.

Children with contraindications to rTMS will be excluded prior to treatment initiation. To monitor fatigue associated with repeated training sessions, the pediatric OMNI Rating of Perceived Exertion scale (0-10) will be administered before each intervention session. Treatment intensity will be modified or sessions postponed if participants report perceived exertion scores ≥7.

All adverse events, including skin irritation, pain, dizziness, nausea, falls, muscle twitching, or seizures, will be recorded using standardized case report forms. Serious adverse events will be reported to the institutional ethics committee within 24 hours and will prompt immediate clinical evaluation and consideration of treatment discontinuation.

#### Treatment Adherence Monitoring

Treatment adherence will be monitored throughout the 8-week intervention period using standardized attendance logs maintained by treating therapists. Attendance will be recorded at each scheduled treatment session and reviewed weekly by the study coordinator.

Adherence will be calculated as the proportion of completed sessions relative to the total number of scheduled sessions. Participants who attend less than 80% of the prescribed intervention sessions will be considered nonadherent and excluded from the per-protocol analysis but retained in the intention-to-treat analysis.

Any missed sessions, reasons for absence, and deviations from the intervention protocol will be documented prospectively. To enhance adherence, guardians will receive regular reminders and appointment confirmations during the study period.

### Outcome Measures

#### Primary Outcomes

The primary outcome of this study is the change in GMFM-88 score from baseline to week 8.

The GMFM-88 is a validated and widely used instrument for evaluating gross motor function in children with CP [18]. It comprises 88 items organized into 5 developmental domains, including lying and rolling; sitting; crawling and kneeling; standing; and walking, running, and jumping. Each item is scored on a 4-point ordinal scale (0-3), with higher scores indicating better motor performance.

The GMFM-88 was selected rather than the GMFM-66 because of its broader item coverage and greater sensitivity to functional change across heterogeneous CP populations with varying Gross Motor Function Classification System (GMFCS) levels.

#### Secondary Outcomes

##### Muscle Tone

Muscle tone will be assessed using the MAS, a commonly used clinical tool for evaluating spasticity [19]. MAS grades (0, I, I+, II, III, and IV) will be converted into ordinal scores (0-5) for statistical analysis, with higher scores indicating greater muscle tone.

##### Gait Performance

Gait performance will be evaluated using a 3D gait analysis system [20]. Reflective markers will be placed at standardized anatomical landmarks, and both static and dynamic gait data will be collected according to institutional gait laboratory protocols.

Participants will perform repeated walking trials at self-selected walking speed along a standardized walkway. Key gait parameters will include gait cycle duration, cadence, stance and swing phase duration, step width, and step length.

##### Electromyography Assessment

Electromyography assessments will be performed using the Nicolet EDX system (Natus Medical Incorporated, Pleasanton). Nerve conduction velocity, latency, and amplitude of the tibial, peroneal, and superficial peroneal nerves will be recorded.

### Sample Size Estimation

Sample size estimation was based on the primary outcome (change in GMFM-88 score at week 8). On the basis of a recent meta-analysis of noninvasive brain stimulation (including rTMS) in children with CP, which reported effect sizes ranging from 0.47 to 0.63 for GMFM outcomes [13], a moderate between-group effect size (Cohen *d*=0.60) was assumed for the primary outcome.

Using G*Power (version 3.1.9.7), with a 2-sided α level of .05 and 80% statistical power, the estimated minimum sample size was 46 participants per group. Allowing for an anticipated attrition rate of 15%, the final recruitment target was set at 54 participants per group, yielding a total sample size of 108.

### Quality Control

#### Design Phase

All investigators and therapists will undergo centralized training before study initiation. Standardized operating procedures will be implemented to ensure consistency in participant screening, intervention delivery, and outcome assessment.

#### Treatment Phase

Weekly multidisciplinary meetings will be conducted to review intervention fidelity, monitor protocol adherence, and address operational issues.

#### Data Management

Study data will be entered independently by 2 trained researchers using a double-entry system. Routine validation checks and discrepancy resolution procedures will be conducted to ensure data accuracy and integrity.

### Statistical Analysis

Statistical analyses will be conducted using SPSS (version 26.0; IBM Corp). A 2-sided α level of .05 will be adopted.

### Descriptive Analysis

Continuous variables will be summarized as means and SDs or medians and IQRs, depending on data distribution assessed using the Shapiro-Wilk test. Categorical variables will be summarized using frequencies and percentages.

#### Primary Estimand

The primary estimand is the between-group difference in the change in GMFM-88 score from baseline to week 8, in the target population of adolescents with spastic CP (GMFCS I-V, age 13-18 years), under the observed treatment conditions without intercurrent event adjustment (treatment-policy estimand).

#### Primary Analysis

The primary analysis will evaluate between-group differences in GMFM-88 change over time using a mixed-effects model for repeated measures. Fixed effects will include treatment group, time, and group-by-time interaction, while participant-level random effects will account for within-subject correlation. An unstructured covariance matrix will be specified.

#### Covariates for Confounding Adjustment

Given the nonrandomized design, the following baseline covariates will be included in multivariable models and propensity score models: age, sex, GMFCS level, baseline GMFM-88 score, baseline MAS grade, and prior rehabilitation history.

#### Propensity Score and Inverse Probability of Treatment Weighting

To further address confounding by indication, propensity score–based inverse probability of treatment weighting (IPTW) will be applied as a sensitivity analysis. Propensity scores will be estimated using a logistic regression model with group allocation as the dependent variable and the covariates listed above as predictors. After IPTW, balance will be assessed using SMDs, with an SMD<0.1 indicating acceptable balance. Covariate balance will be visualized using Love plots.

#### Missing Data Assumptions and Handling

Missing outcome data will be handled under the missing-at-random assumption using multiple imputation with 20 imputed datasets. Complete-case analysis will be conducted as a sensitivity analysis to assess the robustness of results under the missing-completely-at-random assumption. The primary analysis will use the intention-to-treat principle, including all enrolled participants with available baseline data.

#### Sensitivity Analyses

Sensitivity analyses will include (1) a per-protocol analysis excluding participants with attendance below 80%, as documented by standardized treatment attendance logs, and participants who received substantial additional rehabilitation therapies (defined as more than 3 rehabilitation sessions outside the study protocol during the intervention period); and (2) an IPTW-adjusted analysis based on propensity scores as described above.

#### Multiple Comparisons and Subgroup Analyses

False discovery rate correction will be applied to secondary outcomes to reduce type I error associated with multiple testing.

Exploratory subgroup analyses by GMFCS level (I-III vs IV-V) and age (13-15 vs 16-18 years) will be conducted using interaction terms.

### Ethical Considerations

This study will be conducted in accordance with the Declaration of Helsinki and has received approval from the ethics committee of China National Nuclear Corporation 416 Hospital (YJ-2025-020-01).

Written informed consent will be obtained from all participants and/or their legal guardians before enrollment. Participants and guardians will be informed of the study procedures, potential risks, and benefits, and their right to withdraw from the study at any time without consequences to future medical care. Participant confidentiality will be protected through coded identification and secure data storage. Study findings will be disseminated through peer-reviewed scientific journals and academic conferences.

## Results

This study was funded in 2024. Study recruitment began in January 2025 and as of June 2026, enrollment is ongoing; exact recruitment numbers are being verified with the research coordinator. This study has been approved by the ethics committee (YJ-2025-020-01) and supported by grants S2024094 and CDS2018Z005. Outcomes will be assessed at baseline, week 4, and week 8 via 3D gait analysis, electromyography, GMFM-88, MAS. Data collection is expected to be complete in December 2027, and the data will be analyzed with SPSS (IBM Corp). The results are expected to be published in spring 2028. All enrolled participants will undergo an 8-week intervention program consisting of 5 treatment sessions per week, with outcome assessments conducted at baseline, week 4, and week 8.

## Discussion

### Principal Rationale and Expected Contributions

This study aims to evaluate the clinical outcomes associated with pediatric gait rehabilitation robot-assisted training combined with rTMS in adolescents with spastic CP, compared with conventional rehabilitation therapy. Although both robotic gait training and rTMS have demonstrated potential benefits individually, evidence regarding their combined application in pediatric CP rehabilitation remains limited. Existing studies have suggested that robotic rehabilitation may improve gait biomechanics and motor learning through repetitive task-oriented training, while rTMS may facilitate cortical reorganization and enhance corticospinal excitability [21-23]. However, pediatric-specific protocols and multimodal intervention strategies remain insufficiently established.

The proposed intervention integrates peripheral motor training with central neuromodulation, providing a theoretically synergistic rehabilitation framework. Objective assessments, including 3D gait analysis, electromyography, GMFM-88, and MAS, are incorporated to provide a multidimensional evaluation of gait function, motor performance, and muscle tone.

### Interpretation Within the Context of Study Design

This study is designed as a prospective observational controlled study rather than a randomized controlled trial. Consequently, any between-group differences observed should be interpreted as associations rather than causal effects.

To reduce bias associated with nonrandom allocation, multivariable adjustment and propensity score–based methods will be used. Nevertheless, residual confounding due to unmeasured factors cannot be excluded.

Should the intervention group demonstrate superior outcomes, such findings would support the preliminary clinical value of the combined intervention and provide justification for future randomized controlled trials. Conversely, null findings would not necessarily exclude potential benefit but may suggest limited additional effectiveness within the current observational framework.

### Strengths and Limitations

Several strengths of this protocol should be acknowledged. First, the intervention combines robot-assisted gait rehabilitation with rTMS, targeting both peripheral motor execution and central neuroplastic modulation. Second, outcome assessment incorporates objective and multidimensional measures, including gait analysis and electrophysiological evaluation. Third, standardized intervention procedures and predefined statistical adjustment strategies have been incorporated to improve methodological rigor.

However, several limitations should also be recognized. First, the open-label nature of robotic rehabilitation limits participant and therapist blinding and may introduce performance bias. Although assessor blinding will be implemented whenever feasible, subjective assessments may still be influenced.

Second, the follow-up period is limited to 8 weeks. Given the chronic and developmental nature of CP, short-term outcomes may not fully capture sustained functional improvement. Future studies should incorporate longer-term follow-up assessments (eg, 6 or 12 months) to evaluate the durability of any observed improvements.

Third, participant heterogeneity, including differences in age, severity of motor impairment, and cognitive function, may influence treatment responsiveness.

Fourth, the age range of 13 to 18 years was selected primarily for device compatibility and developmental consistency. Consequently, findings may not be generalizable to younger children with CP.

Fifth, this study is conducted at a single tertiary rehabilitation center, which may limit generalizability. Multicenter studies are needed to validate the findings across diverse populations and clinical settings.

Finally, as an observational study, residual confounding and confounding by indication remain possible despite statistical adjustment. Furthermore, differences in intervention structure and treatment frequency between groups may partially influence observed outcomes.

### Comparison With Previous Research and Future Directions

Previous studies investigating rTMS or robotic rehabilitation in CP have primarily evaluated these modalities independently, with limited evidence regarding integrated intervention models. Additionally, stimulation parameters, training intensity, and intervention duration remain heterogeneous across studies, limiting comparability and standardization.

This study may contribute preliminary evidence regarding combined neuromodulation and robotic rehabilitation strategies and help inform future protocol optimization. Future research should prioritize multicenter randomized controlled trials, longer-term follow-up, and mechanistic investigations using neuroimaging and neurophysiological techniques. Integration with emerging technologies, such as virtual reality and biofeedback systems, may further enhance individualized rehabilitation approaches.

### Conclusions

This protocol describes a prospective observational study evaluating the clinical outcomes associated with robot-assisted pediatric gait rehabilitation training combined with rTMS in adolescents with spastic CP.

By integrating objective outcome assessment with multimodal rehabilitation intervention, this study is expected to provide preliminary evidence regarding the feasibility and potential clinical value of combining neuromodulation and robot-assisted therapy in pediatric rehabilitation.

Given the nonrandomized design, findings should be interpreted cautiously and viewed as hypothesis-generating rather than confirmatory. Future randomized and multicenter studies will be necessary to establish causality and optimize intervention strategies.

## Data Availability

Deidentified study data will be stored in a password-protected institutional repository of the Second Affiliated Hospital of Chengdu Medical College and will be available upon reasonable request to the corresponding author after study completion and primary publication. The study protocol and statistical analysis plan will be publicly available via the Chinese Clinical Trial Registry (ChiCTR2500105823).

## Abbreviations

CP: cerebral palsy
GMFCS: Gross Motor Function Classification System
GMFM: Gross Motor Function Measure
IPTW: inverse probability of treatment weighting
MAS: Modified Ashworth Scale
rTMS: repetitive transcranial magnetic stimulation
SMD: standardized mean difference
SPIRIT: Standard Protocol Items: Recommendations for Interventional Trials
STROBE: Strengthening the Reporting of Observational Studies in Epidemiology
